# Evidence-Based Therapeutic Methods for Reflex Sympathetic Dystrophy: A Systematic Review

**DOI:** 10.7759/cureus.115240

**Published:** 2026-08-26

**Authors:** Humera Bagwan, Sandeep Shinde

**Affiliations:** 1 Department of Musculoskeletal Physiotherapy, Krishna College of Physiotherapy, Krishna Vishwa Vidyapeeth, Deemed to be University, Karad, IND; 2 Department of Musculoskeletal Sciences, Krishna College of Physiotherapy, Krishna Vishwa Vidyapeeth, Deemed to be University, Karad, IND

**Keywords:** complex regional pain syndrome, multidisciplinary management, pain management, reflex sympathetic dystrophy, rehabilitation

## Abstract

Complex regional pain syndrome (CRPS) is a chronic pain illness characterized by ongoing discomfort, sensory abnormalities, motor dysfunction, edema, and decreased functional capacity. Although different therapeutic options have been presented, the efficacy of physical rehabilitation and pharmaceutical therapies is still under research. This systematic review sought to assess the existing data on the efficacy of physiotherapy rehabilitation and pharmaceutical therapies in improving pain, function, and quality of life in people with CRPS.

A systematic review of randomized controlled trials (RCTs) published between 2016 and 2024 was conducted. Electronic databases, including PubMed, Scopus, PEDro, Cochrane Library, and Google Scholar, were searched. RCTs published in English that investigated physiotherapy rehabilitation or pharmacological interventions for CRPS were included. Fourteen studies met the eligibility criteria. Methodological quality was assessed using a standardized risk-of-bias assessment tool. Due to heterogeneity among interventions and outcome measures, a narrative synthesis was performed.

Fourteen RCTs met the inclusion criteria. Overall, interventions demonstrated improvements in pain, functional capacity, and quality of life; however, the magnitude and consistency of treatment effects varied substantially across studies. The results imply that current CRPS therapeutic options may improve pain, function, and quality of life; however, treatment efficacy differs between trials. Key obstacles include diverse clinical presentations, a lack of defined treatment methods, and insufficient long-term data. Future research should focus on creating standardized diagnostic criteria, accurate biomarkers, personalized treatment techniques, and large-scale clinical trials to enhance patient outcomes.

The findings indicate that physiotherapy rehabilitation is important in the treatment of CRPS and can successfully improve pain and functional performance. Neridronate appeared to be the most promising pharmaceutical strategy in the research reviewed. However, the heterogeneity in treatment methods, outcome measures, and sample sizes restricts the generalizability of the results. More large-scale, high-quality randomized controlled studies are needed to provide uniform treatment guidelines and maximize clinical outcomes in patients with CRPS.

## Introduction and background

Complex regional pain syndrome (CRPS), historically referred to as reflex sympathetic dystrophy (RSD), is a chronic pain disorder characterized by persistent regional pain that is disproportionate to the intensity or duration of the initial injury [1,2]. Patients may present with a combination of sensory, vasomotor, sudomotor, motor, and trophic abnormalities, including hyperalgesia, allodynia, edema, temperature asymmetry, skin color changes, altered sweating, muscle weakness, restricted range of motion, and changes in the skin, nails, and hair [2,3]. Common precipitating events include fractures, surgical procedures, soft-tissue injuries, sprains, and other forms of trauma, although an identifiable triggering event is not always present. The clinical presentation is heterogeneous and may fluctuate considerably between individuals. Symptoms may persist beyond the expected tissue-healing period and can result in substantial functional limitations, impaired physical performance, reduced independence in daily activities, and diminished quality of life [3].

The conceptualization and classification of CRPS have evolved considerably over the past century. During the American Civil War, the condition was initially described as "causalgia," a term used to characterize severe burning pain associated with nerve injury. Subsequently, the term "reflex sympathetic dystrophy" became widely used because the clinical manifestations were initially attributed primarily to sympathetic nervous system dysfunction [3,4]. Advances in the understanding of chronic pain demonstrated that CRPS involves multiple biological mechanisms beyond sympathetic dysfunction, leading the International Association for the Study of Pain (IASP) to introduce the term "Complex Regional Pain Syndrome" in 1994 [4,5]. CRPS is currently classified into Type I, in which no confirmed nerve injury is identified, and Type II, which occurs following a documented nerve injury. This classification has improved diagnostic consistency and provided a standardized framework for clinical research and therapeutic development [5,6].

CRPS is a rare disorder, with recent evidence reporting a five-year incidence of approximately 0.07% and a prevalence of approximately 26 per 100,000 population [6]. It can impose a substantial clinical and socioeconomic burden on affected individuals. Epidemiological studies indicate a higher incidence among women, with the upper extremity being frequently affected following injury. Persistent pain, restricted mobility, functional impairment, and difficulty performing activities of daily living may interfere with employment, leisure activities, and social participation. The chronic nature of the condition may also contribute to psychological distress, including anxiety, depressive symptoms, fear of movement, and reduced self-esteem. These psychological and social consequences can further influence functional recovery and quality of life. Delayed diagnosis or inadequate management may contribute to persistent disability, increased healthcare utilization, and greater economic burden. Therefore, timely recognition and appropriate management are important for improving functional recovery and long-term outcomes [6,7].

The pathophysiology of CRPS remains incompletely understood and involves interactions among neurological, inflammatory, immune, and vascular mechanisms. Current evidence implicates neurogenic inflammation, peripheral and central sensitization, autonomic dysfunction, immune-mediated responses, and maladaptive neuroplastic changes in the persistence of symptoms [7]. Following tissue injury, inflammatory mediators and neuropeptides may activate peripheral nociceptors, contributing to exaggerated and prolonged pain responses. Alterations in inflammatory cytokines, sympathetic activity, and vascular regulation have also been reported. In addition, changes in cortical organization and body representation may contribute to altered sensory perception and motor dysfunction. Collectively, these mechanisms help explain the heterogeneous clinical manifestations and persistent nature of CRPS [7,8].

Diagnosis remains a significant clinical challenge because no single laboratory test or imaging modality can definitively establish CRPS. Diagnosis requires a comprehensive clinical history and physical examination, together with exclusion of alternative conditions such as peripheral neuropathies, inflammatory disorders, vascular diseases, and musculoskeletal injuries [9]. The Budapest Criteria are currently the most widely accepted diagnostic criteria and provide improved specificity while maintaining high sensitivity compared with earlier diagnostic criteria [9,10]. Nevertheless, diagnostic uncertainty and delayed recognition remain concerns in clinical practice, emphasizing the importance of standardized assessment and early identification [10].

**Figure 1:**
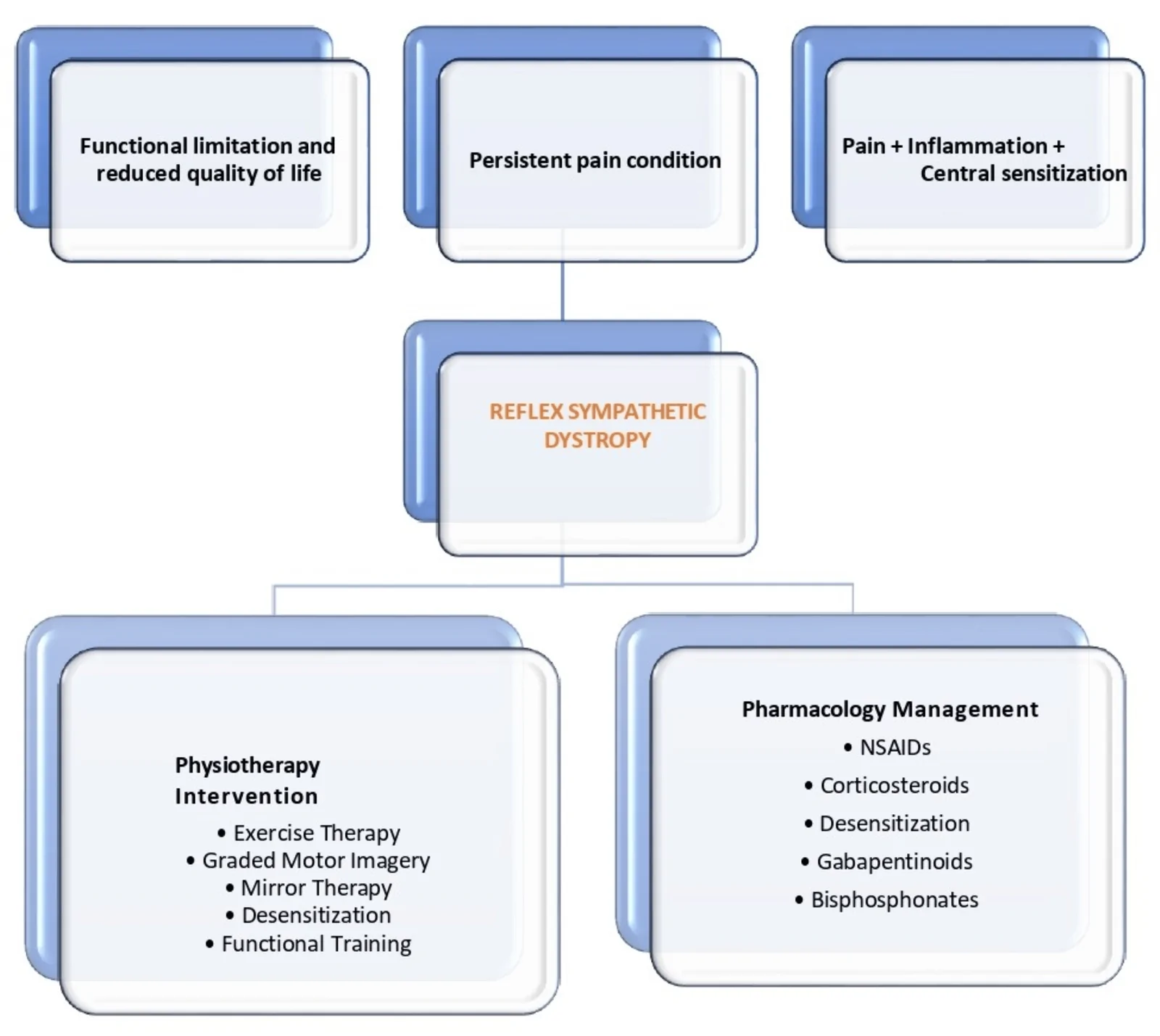
CRPS: Pain Mechanisms, Functional Impact, and Management Approaches CRPS, complex regional pain syndrome; NSAID, non-steroidal anti-inflammatory drug.

Management of CRPS generally requires an individualized and multidisciplinary approach involving rehabilitation, pharmacological treatment, psychological support, and, when indicated, interventional pain management [11,12]. Physiotherapists, physicians, pain specialists, occupational therapists, and psychologists may contribute to coordinated care addressing pain, physical function, activity participation, and psychosocial well-being. Therapeutic approaches include exercise-based rehabilitation, graded motor imagery, mirror therapy, pharmacological interventions, neuromodulation, and emerging technology-assisted strategies such as virtual reality and telerehabilitation. Although several interventions have demonstrated potential benefits, treatment responses remain inconsistent, and evidence varies considerably in methodological quality, outcome measures, sample sizes, and follow-up duration. These limitations, together with the emergence of newer therapeutic approaches, highlight the need for a comprehensive synthesis of current evidence to clarify the effectiveness of available interventions, identify persistent challenges, and guide future research and clinical practice [12] (Figure 1). Therefore, this systematic review aimed to evaluate the current evidence on therapeutic interventions for CRPS, identify challenges in diagnosis and management, examine emerging treatment approaches, and highlight priorities for future research.

## Review

Methodology

Study Design

This systematic review was conducted according to the Preferred Reporting Items for Systematic Reviews and Meta-Analyses (PRISMA) 2020 statement [13]. The review sought to assess the efficacy of physiotherapy rehabilitation and pharmacologic therapies in people with CRPS. Relevant studies were located through systematic database searches, filtered using predetermined eligibility criteria, and critically evaluated to summarize the present evidence.

Literature Search

In April 2026, a comprehensive literature search was conducted across PubMed/MEDLINE, Scopus, PEDro, the Cochrane Library, and Google Scholar. The search strategy combined controlled vocabulary, where available, and free-text keywords related to CRPS, RSD, CRPS Type I, CRPS Type II, physiotherapy, rehabilitation, exercise therapy, mirror therapy, mirror visual feedback, transcutaneous electrical nerve stimulation (TENS), pain exposure physical therapy, fluidotherapy, pharmacological treatment, drug therapy, ketamine, bisphosphonates, neridronate, alendronate, pamidronate, and neuromodulation-related interventions, including spinal cord stimulation and dorsal root ganglion stimulation. Boolean operators (AND and OR) were used to combine search concepts. Additional records were identified by manually screening the reference lists of relevant studies [14]. The complete database-specific search strategies, including the Boolean search strings and applicable filters or limits, are provided in Table 1 to ensure transparency and reproducibility.

**Table 1:** Database-Specific Search Strategies AND, Boolean operator used to combine search terms; CRPS, complex regional pain syndrome; OR, Boolean operator used to broaden the search by including alternative terms; PEDro, physiotherapy evidence database; MEDLINE, Medical Literature Analysis and Retrieval System Online; RCT, randomized controlled trial; RSD, reflex sympathetic dystrophy; TENS, transcutaneous electrical nerve stimulation; TITLE-ABS-KEY, title, abstract, and keyword.

Database	Search strategy (Boolean search string)
PubMed/MEDLINE	("CRPS" OR "Complex Regional Pain Syndrome" OR "Reflex Sympathetic Dystrophy" OR RSD) AND (physiotherapy OR rehabilitation OR "exercise therapy" OR "mirror therapy" OR "mirror visual feedback" OR TENS OR "pain exposure physical therapy" OR fluidotherapy OR pharmacological OR "drug therapy" OR ketamine OR bisphosphonate* OR neridronate OR alendronate OR pamidronate OR neuromodulation OR "spinal cord stimulation" OR "dorsal root ganglion stimulation") AND ("randomized controlled trial" OR randomized OR randomised OR RCT OR trial)
Scopus	TITLE-ABS-KEY("complex regional pain syndrome" OR CRPS OR "reflex sympathetic dystrophy") AND TITLE-ABS-KEY(physiotherapy OR rehabilitation OR exercise OR "mirror therapy" OR "mirror visual feedback" OR TENS OR "pain exposure physical therapy" OR fluidotherapy OR pharmacological OR "drug therapy" OR ketamine OR bisphosphonate* OR neridronate OR alendronate OR pamidronate OR neuromodulation OR "spinal cord stimulation" OR "dorsal root ganglion stimulation") AND TITLE-ABS-KEY(randomized OR randomised OR RCT OR trial)
PEDro	("complex regional pain syndrome" OR "reflex sympathetic dystrophy" OR CRPS) AND (physiotherapy OR rehabilitation OR exercise OR "mirror therapy" OR "mirror visual feedback" OR TENS OR "pain exposure physical therapy" OR fluidotherapy OR pharmacological OR ketamine OR bisphosphonate* OR neuromodulation)
Cochrane Library	("complex regional pain syndrome" OR CRPS OR "reflex sympathetic dystrophy") AND (physiotherapy OR rehabilitation OR exercise OR "mirror therapy" OR "mirror visual feedback" OR TENS OR "pain exposure physical therapy" OR fluidotherapy OR pharmacological OR "drug therapy" OR ketamine OR bisphosphonate* OR neridronate OR alendronate OR pamidronate OR neuromodulation OR "spinal cord stimulation" OR "dorsal root ganglion stimulation") AND (randomized OR randomised OR trial OR RCT)
Google Scholar	"complex regional pain syndrome" OR "reflex sympathetic dystrophy" OR CRPS AND (physiotherapy OR rehabilitation OR exercise OR "mirror therapy" OR "mirror visual feedback" OR TENS OR "pain exposure physical therapy" OR fluidotherapy OR pharmacological OR "drug therapy" OR ketamine OR bisphosphonate* OR neridronate OR alendronate OR pamidronate OR neuromodulation OR "spinal cord stimulation" OR "dorsal root ganglion stimulation")

Selection Criteria

Inclusion criteria: Studies were eligible if they were published in English and involved human participants with CRPS Type I or II. Only randomized controlled trials (RCTs) evaluating the efficacy of physiotherapy rehabilitation interventions and/or pharmacological treatments were included. Eligible studies included participants of any age or gender and reported at least one relevant outcome, including pain, functional capacity, range of motion, edema, disability, grip strength, balance, mobility, or quality of life. Full-text studies providing sufficient methodological information and outcome data relevant to the objectives of the review were included.

Exclusion criteria: Studies were excluded if they were non-English publications, animal studies, laboratory-based experimental studies, conference abstracts without full-text availability, editorials, letters, commentaries, or expert opinion articles. Diagnostic accuracy studies, epidemiological studies, pathophysiological investigations, case reports, case series, and non-randomized studies were also excluded. Studies lacking relevant outcome data, duplicate publications, overlapping datasets, or studies that did not specifically investigate physiotherapy rehabilitation or pharmacological interventions for CRPS were excluded.

Study selection and data extraction: The PRISMA 2020 guidelines were used to screen the studies. A standardized form was used to systematically collect pertinent data, such as research characteristics, outcomes, and major results, from eligible papers after they had undergone full-text evaluation [14,15].

Quality assessment: Methodological quality and risk of bias were assessed using the Cochrane Risk of Bias 2 (RoB 2) tool for RCTs [15,16]. The assessment considered five domains: bias arising from the randomization process, bias due to deviations from intended interventions, bias due to missing outcome data, bias in the measurement of the outcome, and bias in the selection of the reported result. Each domain was assessed according to the RoB 2 guidance, and an overall risk-of-bias judgment was assigned to each included study.

Data synthesis: Because of substantial clinical and methodological heterogeneity among the included studies, a meta-analysis was not considered appropriate. The heterogeneity was related to differences in intervention types, treatment protocols, patient populations, outcome measures, and follow-up periods. Given the substantial differences between interventions, including rehabilitation-based, electrotherapeutic, neurocognitive, and pharmacological approaches, pooling these interventions into a single effect estimate was considered clinically inappropriate and potentially misleading. Therefore, the findings were synthesized narratively and reported in accordance with the principles of the synthesis without meta-analysis reporting guidelines [16]. The included studies were grouped according to the principal intervention category, including physiotherapy and rehabilitation, electrotherapy and physical modalities, neurocognitive and sensorimotor rehabilitation, and pharmacological interventions. Within each intervention category, findings were summarized according to the outcomes reported, including pain, functional performance, range of motion, edema, body perception, grip strength, and quality of life. The direction and consistency of reported treatment effects were compared descriptively across studies, taking into consideration differences in study populations, intervention protocols, outcome measures, and follow-up periods. Study characteristics and key findings were presented in tabular form, while the narrative synthesis was used to describe patterns of benefit, lack of additional benefit, and mixed findings across interventions. No quantitative pooling of effect estimates was performed because the clinical and methodological heterogeneity precluded meaningful statistical synthesis. The limitations associated with the narrative synthesis, including variability in interventions, outcome measures, and follow-up periods, were considered when interpreting the findings.

Results

**Figure 2:**
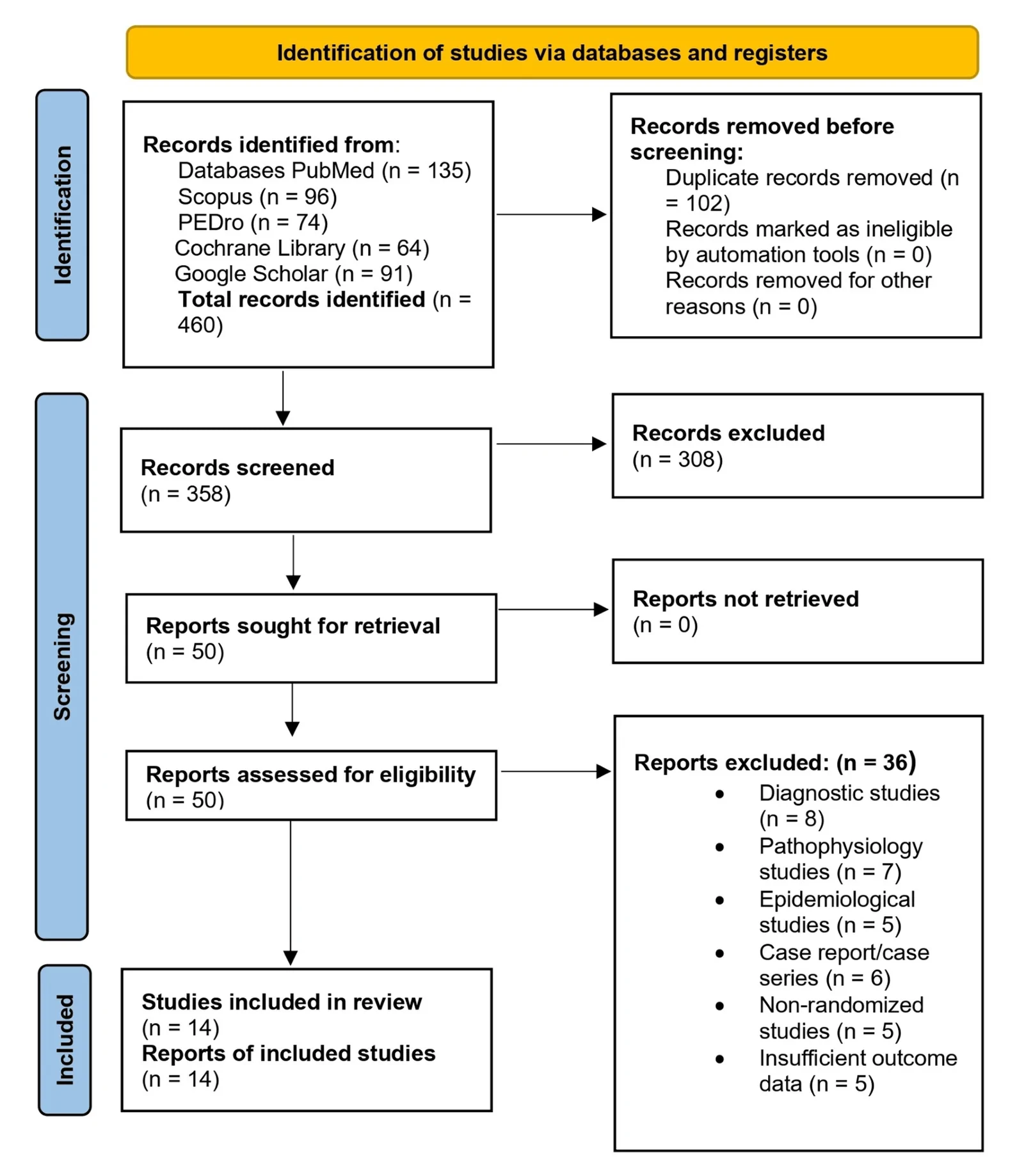
PRISMA Flowchart PRISMA, Preferred Reporting Items for Systematic Reviews and Meta-Analyses.

A systematic literature search was conducted using electronic databases, including PubMed/MEDLINE, Scopus, PEDro, the Cochrane Library, and Google Scholar, with additional records identified through other sources. A total of 460 records were identified, of which 102 duplicates were removed. The remaining 358 records underwent title and abstract screening, resulting in the exclusion of 308 records. Fifty full-text articles were assessed for eligibility, of which 36 were excluded because of diagnostic emphasis, pathophysiological investigations, epidemiological designs, case reports or case series, non-randomized study designs, or inadequate outcome data. Finally, 14 RCTs met the predefined inclusion criteria and were included in the qualitative synthesis [17-30]. The study selection process is presented in the PRISMA 2020 flow diagram (Figure 2).

Fourteen studies published between 2016 and 2024 investigated various treatment approaches for CRPS, including physiotherapy, mirror therapy, electrotherapy, neurocognitive rehabilitation, and pharmacological interventions [17-30]. TENS and fluidotherapy were associated with improvements in pain and functional outcomes. Findings for mirror therapy were mixed across studies. Visual illusion therapy was associated with reductions in pain and body perception disturbance, whereas prism adaptation and pulsed electromagnetic field did not demonstrate significant additional benefits. Among pharmacological interventions, neridronate demonstrated a significant reduction in pain, whereas intravenous immunoglobulin, soticlestat, and nitrous oxide did not demonstrate significant clinical benefits. Detailed study-level findings are presented in Table 2.

**Table 2:** Summary of Evidence-Based Therapeutic Methods for RSD CRPS, complex regional pain syndrome; IVIG, intravenous immunoglobulin; PEMF, pulsed electromagnetic field; RCT, randomized controlled trial; RSD, reflex sympathetic dystrophy; TENS, transcutaneous electrical nerve stimulation.

Sr. no.	Author (year)	Study title	Study design	Sample size	Population	Study category	Key findings
1	Bilgili et al. (2016) [17]	Effectiveness of TENS in patients with CRPS	Randomized double-blind placebo-controlled trial	30	Upper extremity CRPS-I	Electrotherapy	TENS was associated with improvements in pain, edema, range of motion, and functional outcomes compared with the control condition.
2	Vural et al. (2016) [18]	Effects of mirror therapy in stroke patients with CRPS-I	RCT	Not reported	Post-stroke CRPS-I	Mirror therapy	Mirror therapy did not demonstrate an additional benefit compared with routine rehabilitation.
3	Goebel et al. (2017) [19]	Low-dose IVIG treatment for long-standing CRPS	Randomized placebo-controlled trial	111	Chronic CRPS	Immunotherapy	Low-dose IVIG did not demonstrate superiority over placebo for the assessed CRPS outcomes.
4	Barnhoorn et al. (2018) [20]	Pain exposure physical therapy	RCT	56	CRPS-I	Physiotherapy	Pain exposure physical therapy and the comparator intervention produced similar clinical outcomes; lower treatment costs were reported for pain exposure physical therapy.
5	Ozcan et al. (2019) [21]	Effectiveness of fluidotherapy in poststroke CRPS	RCT	30	Post-stroke CRPS-I	Physical modalities	Fluidotherapy was associated with improvements in edema and neuropathic pain.
6	Kotiuk et al. (2019) [22]	The impact of mirror therapy on body schema perception in patients with complex regional pain syndrome after distal radius fractures	RCT	50 (30 intervention, 20 control)	Patients with CRPS-I following distal radius fractures (<3 years duration)	Rehabilitation	Mirror therapy was associated with improvements in body perception, limb awareness, and functional outcomes compared with the control condition.
7	Lewis et al. (2021) [23]	Visual illusions modulate body perception disturbance and pain in CRPS	RCT	45	Upper limb CRPS	Virtual rehabilitation	Visual illusion therapy was associated with reductions in pain and body perception disturbance.
8	Halicka et al. (2021) [24]	Prism adaptation treatment for upper-limb CRPS	Double-blind RCT	49	Upper limb CRPS-I	Neurocognitive rehabilitation	Prism adaptation treatment did not demonstrate a significant benefit for the assessed outcomes.
9	Varenna et al. (2021) [25]	Intramuscular neridronate for CRPS-I	Double-blind placebo-controlled trial	~90	CRPS-I	Pharmacological	Intramuscular neridronate was associated with a significant reduction in pain compared with the control condition.
10	Cömertoğlu et al. (2022) [26]	PEMF therapy in hand CRPS-I	Single-blind RCT	32	Hand CRPS-I	Physical therapy	PEMF did not demonstrate an additional benefit beyond rehabilitation for the assessed outcomes.
11	Ratcliffe et al. (2023) [27]	Soticlestat as adjunctive therapy in adults with CRPS	Phase 2 RCT	24	Chronic CRPS	Pharmacological	Soticlestat did not demonstrate a significant reduction in pain compared with the control condition.
12	Machač et al. (2024) [28]	Mirror visual feedback in CRPS-I	RCT	28	Upper extremity CRPS-I	Mirror therapy	Mirror visual feedback was associated with improvements in pain, grip strength, and quality of life.
13	Özdemir et al. (2024) [29]	Mirror therapy in post-traumatic CRPS-I	RCT	40	Post-traumatic hand CRPS-I	Mirror therapy	Mirror therapy did not demonstrate an additional benefit compared with the control intervention.
14	Hale et al. (2024) [30]	Nitrous oxide for the treatment of CRPS	Randomized blinded trial	44	CRPS type I/II	Pharmacological	Nitrous oxide did not demonstrate a significant improvement in pain or quality of life compared with the control condition.

Assessment of Risk of Bias

The risk-of-bias assessment was conducted for all 14 included RCTs using the Cochrane RoB 2 tool. Five studies, Bilgili et al. [17], Vural et al. [18], Barnhoorn et al. [20], Kotiuk et al. [22], and Hale et al. [30], were judged to have some concerns regarding overall risk of bias, whereas the remaining nine studies, Goebel et al. [19], Özcan et al. [21], Lewis et al. [23], Halicka et al. [24], Varenna et al. [25], Cömertoğlu et al. [26], Ratcliffe et al. [27], Machač et al. [28], and Özdemir et al. [29], were judged to have a low overall risk of bias. No study was judged to have a high overall risk of bias. The domain-level risk-of-bias judgments are presented in Table 3.

**Table 3:** Risk-of-Bias Assessment of Included Randomized Controlled Trials Using RoB 2 RoB 2, Risk of Bias 2.

Sr. no.	Author (year)	Randomization process	Deviations from intended interventions	Missing outcome data	Measurement of outcome	Selection of reported results	Overall risk of bias
1	Bilgili et al. (2016) [17]	Low	Low	Low	Low	Some concerns	Some concerns
2	Vural et al. (2016) [18]	Some concerns	Low	Low	Low	Some concerns	Some concerns
3	Goebel et al. (2017) [19]	Low	Low	Low	Low	Low	Low risk
4	Barnhoorn et al. (2018) [20]	Some concerns	Some concerns	Low	Some concerns	Some concerns	Some concerns
5	Ozcan et al. (2019) [21]	Low	Low	Low	Low	Some concerns	Low risk
6	Kotiuk et al. (2019) [22]	Low	Some concerns	Low	Some concerns	Low	Some concerns
7	Lewis et al. (2021) [23]	Low	Low	Low	Low	Low	Low risk
8	Halicka et al. (2021) [24]	Low	Low	Low	Low	Low	Low risk
9	Varenna et al. (2021) [25]	Low	Low	Low	Low	Low	Low risk
10	Cömertoğlu et al. (2022) [26]	Low	Low	Low	Low	Some concerns	Low risk
11	Ratcliffe et al. (2023) [27]	Low	Low	Low	Low	Low	Low risk
12	Machač et al. (2024) [28]	Low	Low	Low	Low	Low	Low risk
13	Özdemir et al. (2024) [29]	Low	Low	Low	Low	Low	Low risk
14	Hale et al. (2024) [30]	Low	Low	Some concerns	Low	Low	Low risk

Regarding the randomization process, most studies were judged to have a low risk of bias; however, Vural et al. [18] and Barnhoorn et al. [20] raised some concerns in this domain. For deviations from intended interventions, Barnhoorn et al. [20] and Kotiuk et al. [22] were judged to have some concerns, primarily related to blinding and adherence to the intended interventions. Most studies were judged to have a low risk of bias due to missing outcome data, although Hale et al. [30] raised concerns related to participant attrition and incomplete follow-up data.

Regarding bias in the measurement of the outcome, most studies were judged to have a low risk of bias; however, Barnhoorn et al. [20] and Kotiuk et al. [22] raised some concerns related to outcome assessment in the absence of blinding. For selection of the reported result, several studies raised some concerns because of limited information regarding prespecified analysis plans and potential selective reporting. Detailed domain-level judgments for each included study are presented in Table 3.

Discussion

CRPS remains one of the most challenging chronic pain conditions because of its complex etiology, heterogeneous clinical presentation, and variable response to treatment. This systematic review synthesized evidence from 14 RCTs evaluating contemporary therapeutic approaches for CRPS. Overall, several interventions demonstrated potential benefits for pain, functional performance, and quality of life; however, treatment effects varied considerably across studies. This variability reflects the multifactorial nature of CRPS and supports the need for individualized and multidisciplinary management strategies [9,10].

Interventions targeting sensorimotor dysfunction, cortical reorganization, and functional restoration demonstrated promising findings. Improvements in pain, body perception, and functional recovery were reported with mirror therapy, visual illusion therapy, pain exposure therapy, fluidotherapy, and TENS. These findings are consistent with evidence suggesting that maladaptive neuroplasticity, altered body representation, and abnormal central nervous system processing contribute to the persistence of CRPS. However, the effects of these interventions were not uniformly demonstrated across studies. In particular, the mixed findings observed with mirror therapy may be related to differences in treatment duration, frequency, adherence, disease stage, baseline severity, and the ability of individual patients to engage with visual feedback. Such differences in intervention delivery and patient characteristics may contribute to variability in treatment response.

The review also identified limited or negative findings for several pharmacological and adjunctive interventions. The lack of a clear benefit with IVIG may partly reflect the biological heterogeneity of CRPS, as immune-mediated mechanisms may not contribute equally to all patients. Consequently, an intervention targeting immune dysfunction may have greater relevance in selected patient subgroups than in an unselected CRPS population. Similarly, the absence of a significant therapeutic effect with soticlestat suggests that modulation of a single neurobiological pathway may be insufficient to overcome the multiple interacting mechanisms involved in CRPS, including peripheral and central sensitization, neuroinflammation, autonomic dysfunction, and maladaptive neuroplasticity. The limited findings associated with nitrous oxide may also reflect differences in treatment exposure, duration of analgesic effects, and the complex mechanisms underlying persistent CRPS pain. These findings emphasize that therapeutic response may depend on patient phenotype, disease stage, intervention dose and duration, and the biological mechanism targeted by treatment.

Diagnostic uncertainty remains an important challenge in CRPS management. Although the Budapest Criteria have improved diagnostic consistency and remained the most widely accepted clinical framework for CRPS diagnosis, diagnosis continues to rely primarily on clinical history and examination because no single laboratory test, imaging modality, or biomarker can definitively confirm the condition [5,9,11]. Variability in symptom presentation, disease stage, and severity may contribute to delayed recognition or misdiagnosis, particularly because CRPS can share clinical features with neuropathic, inflammatory, vascular, and musculoskeletal conditions. Laboratory investigations and imaging may therefore be useful primarily for excluding alternative diagnoses rather than confirming CRPS. Advances in understanding neuroinflammation, autonomic dysfunction, vascular alterations, and central nervous system changes have generated interest in potential objective diagnostic markers; however, reliable and clinically validated biomarkers have not yet been established. Further research aimed at developing and validating objective diagnostic tools may improve diagnostic accuracy, facilitate earlier recognition, and support more individualized treatment strategies.

Substantial treatment heterogeneity was also evident across the included studies. Intervention protocols, treatment duration, outcome measures, and follow-up periods varied considerably, limiting direct comparison of findings and complicating the development of standardized evidence-based treatment recommendations [17,30]. Several studies also included relatively small samples and short follow-up periods, limiting conclusions regarding long-term treatment effectiveness [11,29]. Although some interventions demonstrated promising effects, differences in treatment intensity, intervention delivery, patient characteristics, and outcome assessment may partly explain the variability in reported results.

The findings further support the importance of a multidisciplinary approach to CRPS management. Effective care may require integration of physical rehabilitation, pain management, psychological support, patient education, and functional restoration. Given the interaction among biological, psychological, and social factors, a biopsychosocial approach may provide a comprehensive framework for addressing the multidimensional consequences of CRPS. Early intervention and individualized treatment planning may be particularly important for preventing persistent disability and promoting functional recovery.

Future research should focus on identifying patient characteristics and biological phenotypes that may predict treatment response. Advances in understanding neuroplasticity, central sensitization, neuroinflammation, and pain modulation may facilitate the development of more targeted interventions. The identification of reliable biomarkers may improve diagnostic accuracy and facilitate earlier treatment initiation. Emerging approaches, including neuromodulation, virtual reality-based rehabilitation, and individualized treatment strategies, also warrant further investigation. Large, adequately powered multicenter RCTs using standardized intervention protocols, clinically meaningful outcome measures, and sufficiently long follow-up periods are needed to establish the effectiveness and long-term clinical relevance of existing and emerging therapies [25,30].

The methodological findings should also be considered when interpreting the results. The risk-of-bias assessment indicated that most included trials had a low overall risk of bias, although some studies raised concerns in specific methodological domains, including the randomization process, deviations from intended interventions, missing outcome data, outcome measurement, and selection of the reported result [10,15]. The predominance of low-risk trials provides some support for the methodological credibility of the available evidence; however, the identified concerns should be considered when interpreting the findings. These methodological considerations, together with variation in intervention protocols, outcome measures, and follow-up periods, may influence the strength and generalizability of the available evidence. Future RCTs should incorporate rigorous randomization procedures, prespecified analysis plans, appropriate outcome assessment procedures, standardized intervention protocols, validated outcome measures, and sufficiently long follow-up periods to strengthen the evidence base and support more consistent clinical recommendations for the long-term management of CRPS.

Clinical Implications

The findings of this systematic review support the importance of early recognition, timely management, and individualized care for individuals with CRPS. A multidisciplinary, patient-centered approach integrating rehabilitation, appropriate pharmacological management, patient education, and functional restoration may help address the multidimensional effects of CRPS. However, the variability in intervention protocols, outcome measures, and treatment responses indicates that therapeutic decisions should be individualized according to patient characteristics, clinical presentation, disease stage, and treatment goals. Consistent assessment methods and evidence-based treatment strategies may support clinical decision-making and optimize functional outcomes [5,26].

Limitations

This systematic review has several limitations. Substantial heterogeneity was observed across the included studies in terms of intervention protocols, treatment duration, outcome measures, patient characteristics, and follow-up periods, which limited direct comparison of findings and precluded quantitative pooling through meta-analysis. Several included trials had relatively small sample sizes, which may limit the statistical power and generalizability of their findings. Some studies also demonstrated concerns in specific risk-of-bias domains, including the randomization process, deviations from intended interventions, outcome measurement, missing outcome data, and selection of the reported result. In addition, the relatively short follow-up periods in several studies limited conclusions regarding the long-term effectiveness and sustainability of treatment effects. These limitations should be considered when interpreting the overall findings and applying them to broader CRPS populations [15,17-30].

## Conclusions

CRPS remains a complex and challenging condition associated with persistent pain, sensory disturbances, motor dysfunction, and substantial functional limitations that can significantly impair quality of life. Current evidence suggests that physiotherapy rehabilitation and selected pharmacological interventions may improve pain, functional performance, and other clinically relevant outcomes, although treatment responses vary considerably because of the heterogeneous nature of the condition. These findings highlight the importance of an individualized, evidence-based approach to CRPS management that considers the patient's clinical presentation, functional limitations, and treatment needs. Early recognition, timely intervention, and a multidisciplinary, patient-centered treatment strategy integrating rehabilitation with appropriate medical care may support functional recovery and improve quality of life. However, substantial heterogeneity in intervention protocols, outcome measures, sample sizes, and follow-up periods limits the generalizability and long-term interpretation of the available evidence. Further adequately powered, high-quality RCTs using standardized intervention protocols, clinically meaningful outcome measures, and sufficiently long follow-up periods are needed to strengthen the evidence base and establish more consistent treatment recommendations for CRPS.
